# Influence of oversimplifying the head anatomy on cerebral blood flow measurements with diffuse correlation spectroscopy

**DOI:** 10.1117/1.NPh.10.1.015010

**Published:** 2023-03-30

**Authors:** Hongting Zhao, Erin M. Buckley

**Affiliations:** a Georgia Institute of Technology and Emory University, Wallace H. Coulter Department of Biomedical Engineering, Atlanta, Georgia, United States; b Emory University School of Medicine, Department of Pediatrics, Atlanta, Georgia, United States; c Children’s Healthcare of Atlanta, Children’s Research Scholar, Atlanta, Georgia, United States

**Keywords:** cerebral blood flow, diffuse correlation spectroscopy, multilayer model

## Abstract

**Significance:**

Diffuse correlation spectroscopy (DCS) is an emerging optical modality for non-invasive assessment of an index of regional cerebral blood flow. By the nature of this noninvasive measurement, light must pass through extracerebral layers (i.e., skull, scalp, and cerebral spinal fluid) before detection at the tissue surface. To minimize the contribution of these extracerebral layers to the measured signal, an analytical model has been developed that treats the head as a series of three parallel and infinitely extending slabs (mimicking scalp, skull, and brain). The three-layer model has been shown to provide a significant improvement in cerebral blood flow estimation over the typically used model that treats the head as a bulk homogenous medium. However, the three-layer model is still a gross oversimplification of the head geometry that ignores head curvature, the presence of cerebrospinal fluid (CSF), and heterogeneity in layer thickness.

**Aim:**

Determine the influence of oversimplifying the head geometry on cerebral blood flow estimated with the three-layer model.

**Approach:**

Data were simulated with Monte Carlo in a four-layer slab medium and a three-layer sphere medium to isolate the influence of CSF and curvature, respectively. Additionally, simulations were performed on magnetic resonance imaging (MRI) head templates spanning a wide-range of ages. Simulated data were fit to both the homogenous and three-layer model for CBF. Finally, to mitigate the errors in potential CBF estimation due to the difficulty in defining layer thickness, we investigated an approach to identify an equivalent, “optimized” thickness via a pressure modulation.

**Results:**

Both head curvature and failing to account for CSF lead to significant errors in the estimation of CBF. However, the effect of curvature and CSF on relative changes in CBF is minimal. Further, we found that CBF was underestimated in all MRI-templates, although the magnitude of these underestimations was highly influenced by small variations in the source and detector optode positioning. The optimized thickness obtained from pressure modulation did not improve estimation accuracy of CBF, although it did significantly improve the estimation accuracy of relative changes in CBF.

**Conclusions:**

In sum, these findings suggest that the three-layer model holds promise for improving estimation of relative changes in cerebral blood flow; however, estimations of absolute cerebral blood flow with the approach should be viewed with caution given that it is difficult to account for appreciable sources of error, such as curvature and CSF.

## 1 Introduction

Adequate cerebral blood flow (CBF) ensures delivery of oxygen and required substrates to maintain normal brain function. Assessment of CBF can aid in the diagnosis and management of numerous conditions, including stroke and traumatic brain injury. Several techniques exist to assess CBF, including perfusion magnetic resonance imaging (MRI), computed tomography, and transcranial Doppler ultrasound. Drawbacks of these approaches include the need for patient transport, exposure to ionizing radiation, and/or lack or microvascular sensitivity. Diffuse correlation spectroscopy (DCS) is an emerging optical tool that quantifies an index of regional microvascular cerebral blood flow. This approach is especially well suited for continuous, beside monitoring,^1^^,^^2^ given its high temporal resolution (∼1 to 100 Hz), use of non-ionizing radiation, relatively low cost (< $50 k), and portability.

In DCS, near-infrared light is injected into the tissue surface and detected some distance away (typically 1 to 3 cm). Red blood cell motion induces temporal fluctuations in the detected light intensity.^3^ A simple analytical model is used to relate these intensity fluctuations to an index of blood flow (BFI, ${\mathrm{cm}}^{2}/s$) of the underlying tissue.^4^ However, by the nature of this noninvasive measurement, light must pass through extracerebral layers (i.e., skull, scalp, and/or cerebral spinal fluid) before detection at the tissue surface. Thus, the BFI measured by DCS reflects a combination of both cerebral and extracerebral hemodynamics.

Multiple methods have been proposed to minimize extracerebral contributions to BFI and to improve brain sensitivity, including both hardware^5^^–^^9^ and novel analytical approaches.^10^^,^^11^ Analytical approaches are particularly attractive because they can be integrated into the analysis strategies of any hardware approach. Among those approaches, a model that treats the head as a series of three parallel, infinitely extending slabs (mimicking scalp, skull, and brain) has been shown to provide a significant improvement in CBF estimation over the traditional DCS analysis approach that treats the head as a bulk homogenous medium.^12^^–^^14^ Although a handful of studies have utilized this three-layer model to analyze *in vivo* data,^12^^,^^13^^,^^15^ the accuracy of this approach is still under investigation.^16^^,^^17^ While it certainly provides a more sophisticated representation of the human head compared to the homogenous model, the three-layer model is still a gross oversimplification. It ignores head curvature, the presence of cerebrospinal fluid (CSF), and heterogeneity in layer thickness. These parameters are important to account for, as they can vary significantly across subjects. Curvature changes dramatically through adolescence as the head circumference undergoes rapid growth. Moreover, the thickness of the CSF layer increases through early adolescence^18^ and then further increases in old age as the brain begins to shrink.^19^^,^^20^ Finally, layer thickness can be highly heterogenous as a function of optode positioning on the head.^18^^,^^21^^–^^24^ Thus, given the high variability in the structure of the human head, a rigorous investigation into the influence of these parameters on estimation accuracy of cerebral blood flow assessed with the three-layer model is needed.

In this study, we quantify the influence of head curvature and the CSF layer on the estimation accuracy of cerebral blood flow with the three-layer model. We hypothesize that both of these factors will confound the absolute value of cerebral blood flow index (CBFi) but not the estimation accuracy of relative changes of CBFi (rCBFi). Further, we use Monte Carlo (MC) simulations on MRI head templates spanning a wide age range to demonstrate the cumulative influence of these factors, along with the influence of heterogeneity in layer thickness. Finally, we investigate a pressure modulation process designed to overcome the difficulties of defining layer thickness *in vivo*.

## 2 Methods

### 2.1 Influence of CSF

#### 2.1.1 Data simulation

**Fig. 1:**
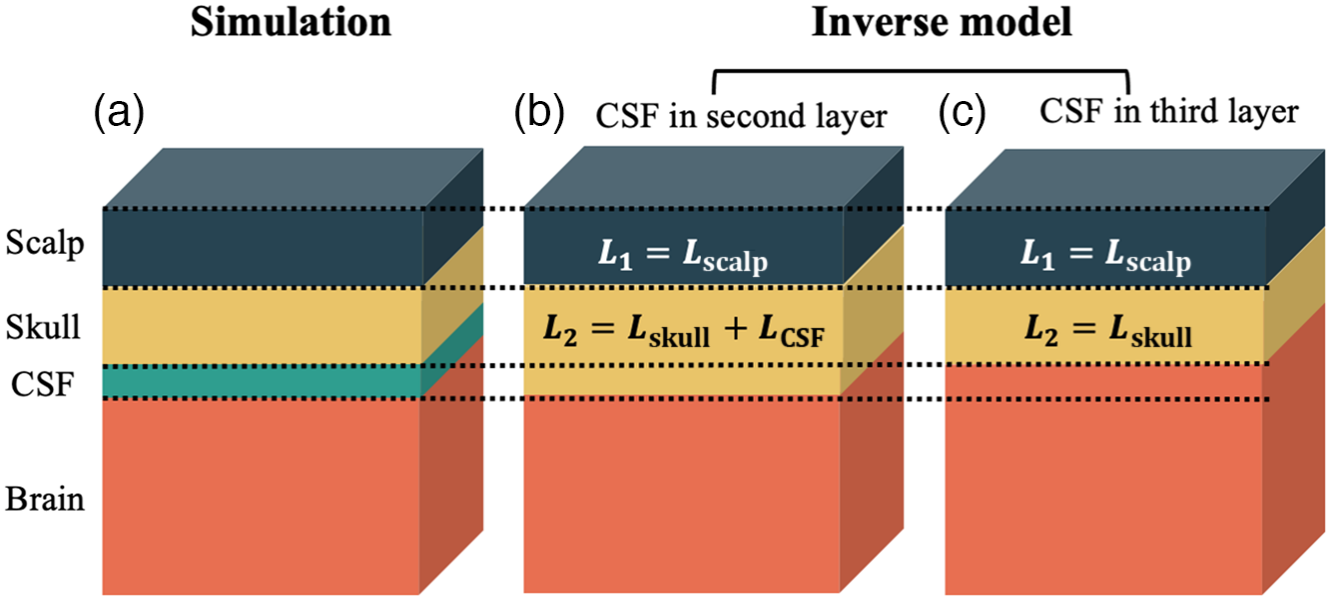
Scheme for investigating the influence of cerebral spinal fluid (CSF) on the accuracy of CBFi with the three-layer model. (a). Data were simulated with MC on a four-slab medium to mimic scalp (blue), skull (yellow), CSF (green), and brain (orange) layers. Simulated data were fit to the three-layer solution of the correlation diffusion equation by assuming CSF belonged either in (b) the second layer ($L_{2}=L_{\text{skull}}+L_{\mathrm{CSF}}$) or (c) the third layer ($L_{2}=L_{\text{skull}}$).

To determine the influence of CSF on the estimation of CBFi obtained with the three-layer model, data were simulated with MC eXtreme (MCX)^25^ on a four-layered slab medium mimicking scalp, skull, CSF, and brain [Fig. 1(a)]. Four separate media were simulated, each of different CSF thickness (1, 2, 3, or 4 mm). All other layer thicknesses, as well as optical properties were constant across simulations: scalp thickness was 6 mm, skull thickness was 6 mm to simulate the frontal region of a healthy young adult,^24^ and the optical properties (i.e., the absorption and reduced scattering coefficient, $\mu_{a}$ and $\mu_{s}^{\prime }$) of each layer were fixed at $\mu_{a,\text{scalp}}=0.1\;{\mathrm{cm}}^{-1}$, $\mu_{s,\text{scalp}}^{\prime }=10\;{\mathrm{cm}}^{-1}$, $\mu_{a,\text{skull}}=0.1\;{\mathrm{cm}}^{-1}$, $\mu_{s,\text{skull}}^{\prime }=10\;{\mathrm{cm}}^{-1}$, $\mu_{a,\mathrm{CSF}}=0.04\;{\mathrm{cm}}^{-1}$, $\mu_{s,\mathrm{CSF}}^{\prime }=0.036\;{\mathrm{cm}}^{-1}$, $\mu_{a,\text{brain}}=0.15\;{\mathrm{cm}}^{-1}$, and $\mu_{s,\text{brain}}^{\prime }=4\;{\mathrm{cm}}^{-1}$.^19^^,^^24^^,^^26^^–^^29^ The anisotropic factor (g) and index of refraction (n) of each layer were fixed at 0.89 and 1.4, respectively.

For each simulation, $10^{9}$ photons were injected into the medium. Two 1-mm diameter detectors were placed on the surface of the top (scalp) layer, spaced 1 and 2.5 cm from the source. For each detected photon, MCX records the momentum transfer, scattering angle, and total pathlength traveled in each layer. This information is used to calculate the unnormalized electric field autocorrelation function $G_{1}(\rho ,\tau )$ by assuming a blood flow index in each layer.^3^ We simulated 12 evenly spaced CBFi values $\in [2,9]\times 10^{-8}\;{\mathrm{cm}}^{2}/s$ and 6 scalp blood flow index (SBFi) values ∈[1/8,1/3]×CBFi, for a total of 72 combinations of SBFi and CBFi per simulation. CSF was modeled as a low-scattering medium with negligible ($1\times 10^{-10}\;{\mathrm{cm}}^{2}/s$) blood flow index.^30^^,^^31^ Similarly, blood flow index of the skull layers was also set to $1\times 10^{-10}\;{\mathrm{cm}}^{2}/s$.^24^ Finally, to simulate experimental measurements, $G_{1}(\rho ,\tau )$ was converted to $g_{2}(\rho ,\tau )$ using the Siegert relation by assuming a coherence factor, β, of 0.5.^2^

#### 2.1.2 Cerebral blood flow estimation

Simulated data at 1 and 2.5 cm were simultaneously fit to the three-layer solution of the correlation diffuse equation (CDE) to estimate the blood flow index of the brain and scalp layers (CBFi and SBFi, respectively).^16^^,^^17^ For these fits, optical properties for each layer were assumed to be known and set to the properties listed above. Thickness of the top layer was set to the known thickness of the scalp (6 mm). For the thickness of second layer, we considered two cases. In the first case, CSF was assumed to be part of skull (layer 2), as CSF has negligible blood flow akin to skull. Thus, the thickness of the second layer, $L_{2}$, was $L_{\text{skull}}+L_{\mathrm{CSF}}$, where $L_{\text{skull}}$ is the thickness of skull and $L_{\mathrm{CSF}}$ is the thickness of CSF. [Fig. 1(b)]. In the second case, CSF was ignored and lumped with the brain because of its transparency. Thus, the thickness of the second layer was set to that of skull [6 mm, Fig. 1(c)]. In both cases, the blood flow index in the second layer was assumed to be zero.

### 2.2 Influence of Curvature

#### 2.2.1 Data simulation

**Fig. 2:**
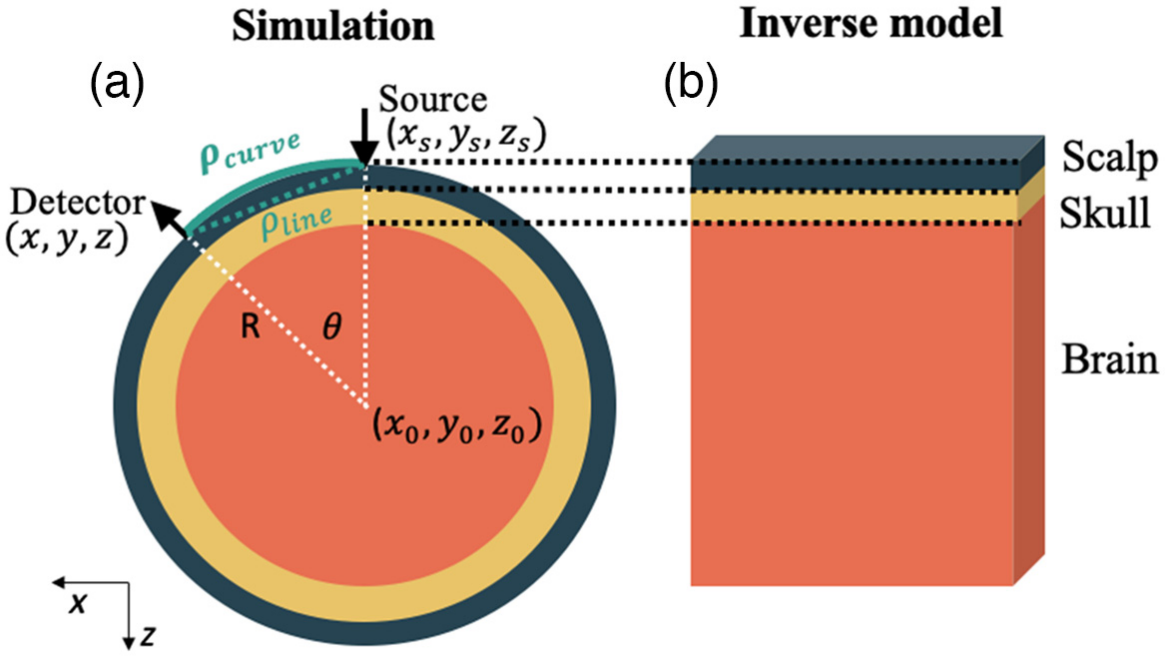
Scheme for investigating the influence of curvature on the accuracy of with the three-layer model. (a) Data were simulated with MC on a three-layer sphere. $\rho_{\text{curve}}$ denotes the arc distance (green curve) along the surface from the source to the detector while $\rho_{\text{line}}$ denotes the linear distance. (b) Simulated data were fit to the three-layer solution of the correlation diffusion equation.

To determine the influence of head curvature on the estimation of CBFi with the three-layer model, data was simulated using a mesh-based MC^32^ on a three-layer sphere medium, mimicking scalp, skull, and brain layers. Four separate curvatures were simulated by varying the radius of the outer sphere from 70 to 100 mm in steps of 10 mm.^33^ These radii were chosen by approximating the head as a circle and extrapolating from normal human head circumference ranges from 1 year to adulthood. Layer thickness and optical properties were constant across simulations and set to the values listed in Sec. 2.1. Detectors were placed along the surface of the outer sphere, spaced 1 and 2.5 cm from the source [$\rho_{\text{curve}}$ in Fig. 2(a)]. As described in Sec. 2.1, we simulated 72 combinations of SBFi and CBFi for each radius.

#### 2.2.2 Cerebral blood flow estimation

Simulated data were fit to the three-layer solution of the CDE to estimate CBFi and SBFi.^16^^,^^17^ For these fits, optical properties and thickness of each layer were assumed to be known and equal to the true (simulated) value. To confirm the error in estimation of CBFi is caused by the influence of curvature rather than by error in source detector separation (SDS) caused by curvature, we fit data using both the line SDS [$\rho_{\text{line}}$ in Fig. 2(a)] and $\rho_{\text{curve}}$.

### 2.3 Realistic Human Head Geometry

#### 2.3.1 Data simulation

**Fig. 3:**
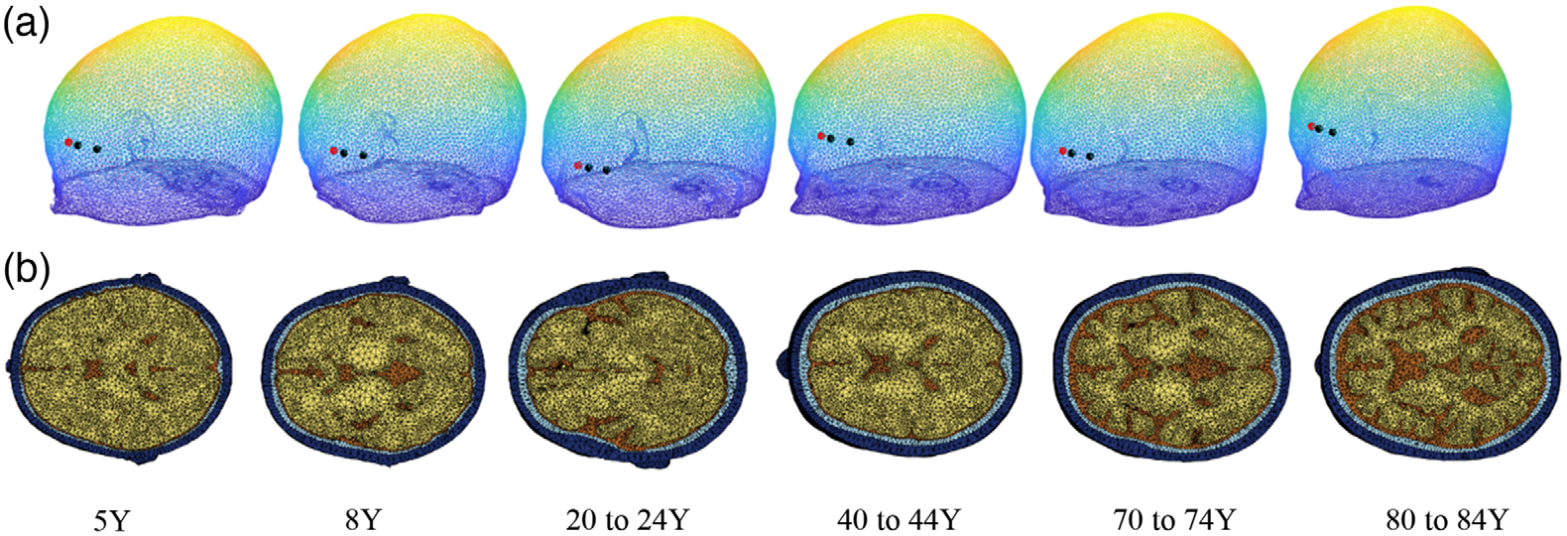
Age-averaged MRI templates. (a) 3D mesh of the contour of each atlas along with the source (red) and detectors locations (black). (b) Axial view of the plane where source and detectors were placed (scalp in dark blue, skull in light blue, CSF in brown, and brain in yellow).

To quantify the accuracy CBFi estimated with the three-layer model in the presence of CSF, head curvature, and heterogeneous layer thicknesses, we simulated data with a mesh-based MC^32^ on six MRI head templates spanning a wide age range of 5 to 80 years (Fig. 3).^34^ We limited simulations to >5 years because the homogeneous model has been shown to be sufficient for younger children given the relatively thin extracerebral layers.^35^^,^^36^ Age-averaged MRI templates were obtained from the open-source brain mesh library.^37^^,^^38^ Templates were segmented into scalp, skull, CSF, gray matter, and white matter layers. For simplicity, gray and white matters were lumped together as brain. Optical properties of each layer were assigned as in Sec. 2.1.

For these simulations, a source and two detectors were placed over the left forehead in the same axial plane, roughly 1 cm above the eyebrow and spaced ∼1 and 2.5 cm apart. Detected photons were used to estimate $g_{2}(\tau ,\rho )$ for a range of 72 simulated SBFi and CBFi, as in Sec. 2.1.

#### 2.3.2 Cerebral blood flow estimation with the 3-layer model

Simulated data at 1 and 2.5 cm were simultaneously fit to the three-layer model to estimate CBFi and SBFi.^16^^,^^17^ For these fits, optical properties of each layer were assumed to be known and equal to the true (simulated) value. Layer thickness for these simulations was challenging to determine because of heterogeneities under the region spanned by the optodes. Thus, we explored two approaches to assess the layer thicknesses.

1. Volume-averaged thickness: because photons propagate through a three-dimensional region under the source/detector, we measured layer thickness in 11 axial slices (5 slices below the S-D plane, the slice containing the S-D plane, and 5 slices above the S-D plane). Slices were spaced 2 mm apart, thus, the total interrogated region spanned ±1  cm above/below the S-D plane. For each slice, we used the method outlined in Ref. 24 to estimate thickness. For each node on the surface of scalp between the x- and y-locations of the source and 2.5-cm detector, the depth to each layer (i.e., skull, CSF, and brain) was defined as the shortest distance between the scalp node and the nearest layer node. Scalp ($L_{1}$) thickness was defined as the depth to the skull layer; skull thickness ($L_{2}$) was calculated depth to CSF minus depth to skull; and CSF thickness was calculated as depth to brain minus depth to CSF. Finally, $L_{1,\text{measured}}$ and $L_{2,\text{measured}}$ were estimated by averaging across all nodes and all slices.
2. Pressure-optimized thickness: an alternative approach to estimate layer thicknesses involves a brief pressure modulation.^13^ Here we assume that applying pressure to the scalp decreases SBFi but does not change CBFi. To simulate this procedure, for each MRI template we simulated two sets of $g_{2}(\tau )$ at 1 and 2.5 cm to mimic the pressure on/off conditions. For these simulations, $\mathrm{CBF}i_{\text{off}}={\mathrm{CBFi}}_{\text{on}}=5.2\times 10^{-8}\;{\mathrm{cm}}^{2}/s$, ${\mathrm{SBFi}}_{\text{off}}=1.7\times 10^{-8}\;{\mathrm{cm}}^{2}/s$, and ${\mathrm{SBFi}}_{\text{on}}=6.5\times 10^{-9}\;{\mathrm{cm}}^{2}/s$. The choice of a 60% decrease inscalp flow from the pressure off-to-on condition comes from *in vivo* observations of BFI changes at 1 cm with gentle pressure.
  Next, simulated $g_{2,\text{off}}$ at 1 and 2.5 cm were simultaneously fit for ${\mathrm{SBFi}}_{\text{off}}$ and ${\mathrm{CBFi}}_{\text{off}}$. For these fits, we assumed known layer optical properties, along with a range of layer thickness ($L_{1}\in [L_{1,\text{measured}}-0.2,L_{1,\text{measured}}+0.2]\;\mathrm{cm}$, $L_{2}\in [L_{2,\text{measured}}-0.2,L_{2,\text{measured}}+0.2]\;\mathrm{cm}$, step size = 0.05 cm) to estimate ${\mathrm{CBFi}}_{\text{off},\text{fit}}(L_{1},L_{2})$. A similar process was applied to $g_{2,\text{on}}$ to estimate ${\mathrm{CBFi}}_{\text{on},\text{fit}}(L_{1},L_{2})$. Last, we defined the optimized thicknesses, $L_{1}$ and $L_{2}$, as those that minimized the relative change in CBFi.
  $$L_{1,\text{optimized}},L_{2,\text{optimized}}={\mathrm{argmin}}_{L_{1},L_{2}}(|\frac{{\mathrm{CBFi}}_{\text{on},\text{fit}}(L_{1},L_{2})}{{\mathrm{CBFi}}_{\text{off},\text{fit}}(L_{1},L_{2})}-1|).$$

#### 2.3.3 Cerebral blood flow estimation using the homogeneous model

To compare the three-layer model to the traditionally employed homogenous model, we also fitted MRI simulated data with this more commonly utilized approach. For these fits, optical properties were assumed to be $\mu_{a}=0.15\;{\mathrm{cm}}^{-1}$ and $\mu_{s}^{\prime }=4\;{\mathrm{cm}}^{-1}$.

### 2.4 Evaluations of Estimation Accuracy of CBFi

We calculated the percentage error in estimated CBFi as $({\mathrm{CBFi}}_{\mathrm{est}}-{\mathrm{CBFi}}_{\text{known}})/{\mathrm{CBFi}}_{\text{known}}\times 100$. Further, because we are often clinically interested in assessing relative changes in CBF as a function of time, we quantified relative changes in CBFi. For each simulated CBFi value, we defined the relative change in CBFi as $\mathrm{rCBFi}=(\mathrm{CBFi}-{\mathrm{CBFi}}_{0})/{\mathrm{CBFi}}_{0}$, where the subscript 0 denotes a baseline/reference measurement, which was arbitrarily chosen to be ${\mathrm{CBFi}}_{0}=5.18\times 10^{-8}\;{\mathrm{cm}}^{2}/s$ and $SBFi_{0}=1.04\times 10^{-8}\;{\mathrm{cm}}^{2}/s$. This baseline ensured a wide range of simulated rCBFi (−61 to 74%) and rSBFi (−76 to 189%). Error in estimated rCBFi was defined as ${\mathrm{rCBFi}}_{\mathrm{est}}-{\mathrm{rCBFi}}_{\text{known}}$.

## 3 Results

### 3.1 Influence of Cerebrospinal Fluid

**Fig. 4:**
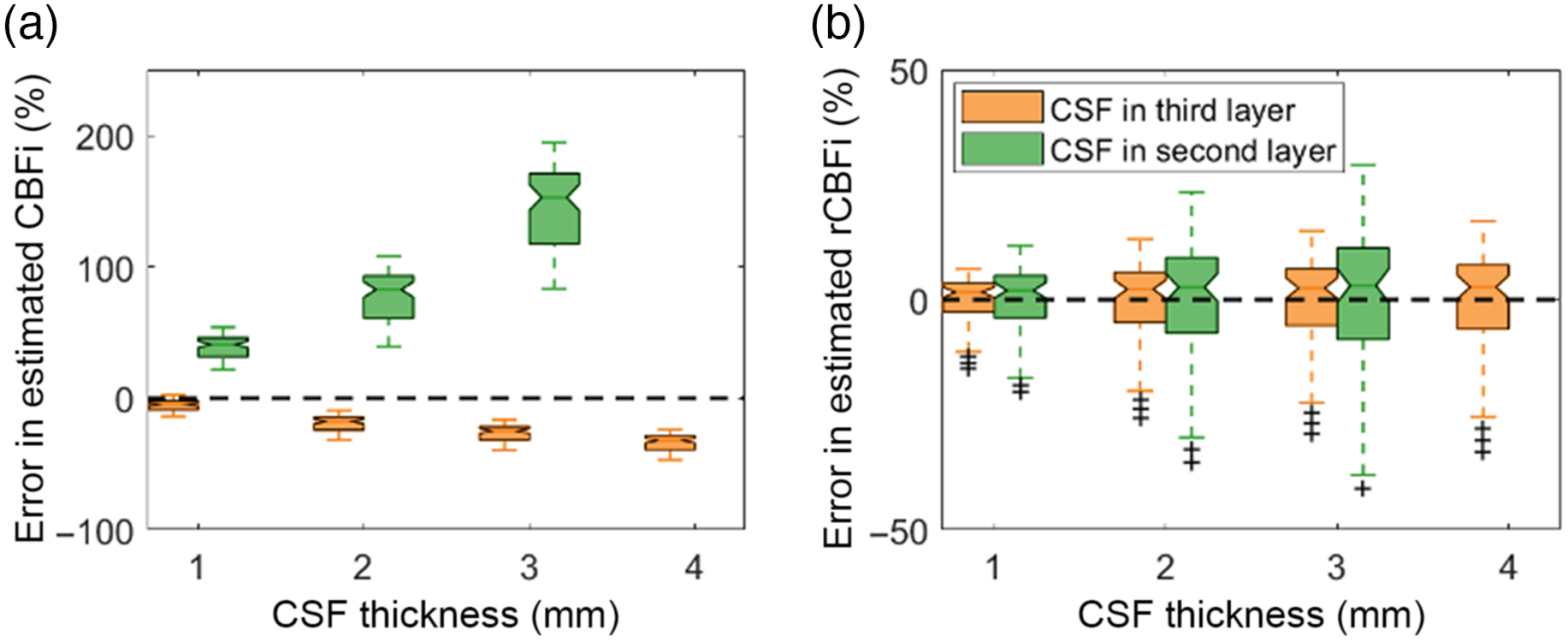
Influence of CSF. Boxplots showing the percentage error in estimated CBFi (a) and relative changes in CBFi (rCBFi) (b) when considering CSF as part of second (green) or third (orange) layer during the fitting process. For each boxplot, the central line denotes the median, and the bottom and top edges of the box indicate the 25’th and 75’th percentiles, respectively, of the 72 samples tested. The whiskers extend to the most extreme data points not considered outliers.

Grouping CSF into the second layer leads to large errors in the estimation of CBFi [Fig. 4(a), green]. As CSF thickness increases, CBFi is significantly overestimated. Note, when CSF thickness was 4 mm, the fitting process failed to converge. In contrast, ignoring CSF, i.e., grouping CSF with the third layer, leads to underestimation in CBFi [Fig. 4(a), orange]. In this situation, as the thickness of CSF increases, the error in CBFi decreases monotonically, although the magnitude of this error is considerably less that the case where CSF is grouped with the skull layer. Relative changes in CBFi can be accurately recovered in both models to within approximately 10%; however, the range of error in estimated rCBFi across all combinations of CBFi and SBFi tested is smaller when CSF is grouped in the third layer compared to the second layer [Fig. 4(b)].

### 3.2 Influence of Head Curvature

**Fig. 5:**
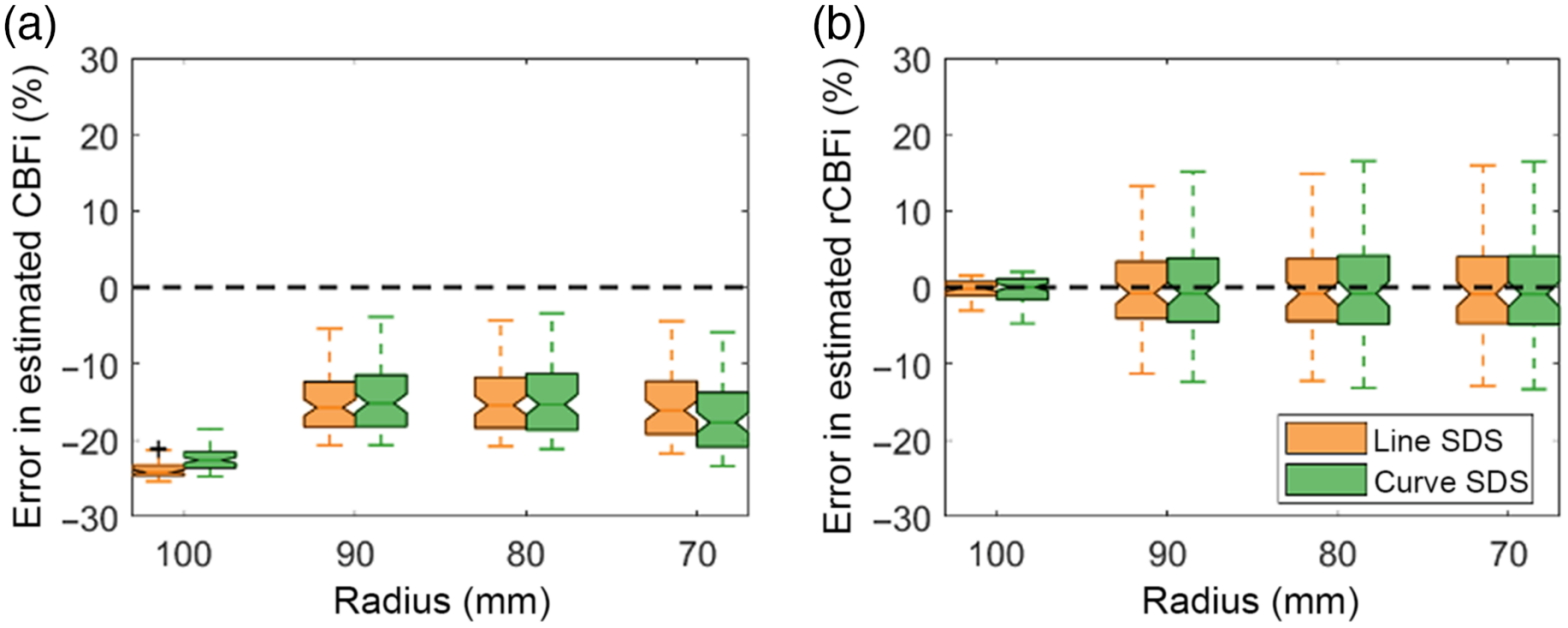
Influence of curvature. Boxplots of the percentage error in estimated CBFi (a) and relative changes in CBFi (rCBFi) (b) when using the three-layer analytical model with the arc distance along the surface from the source to the detector (green) or the linear distance between source and detector (orange). For each boxplot, the central line denotes the median, and the bottom and top edges of the box indicate the 25’th and 75’th percentiles, respectively, of the 72 samples tested. The whiskers extend to the most extreme data points not considered outliers.

Across all simulated head circumferences, curvature led to a median underestimation of CBFi of roughly −10 to −25% [Fig. 5(a)]. This error was relatively independent of circumference. Similarly, the choice of source–detector separation (line versus curve) had minimal influence on this underestimation [orange versus green, Fig. 5(a)]. Moreover, errors in rCBFi caused by curvature were within 15% of the true value across all SBFi/CBFi combinations tested [Fig. 5(b)].

### 3.3 Accuracy of Three-Layer Model as a Function of Age

**Fig. 6:**
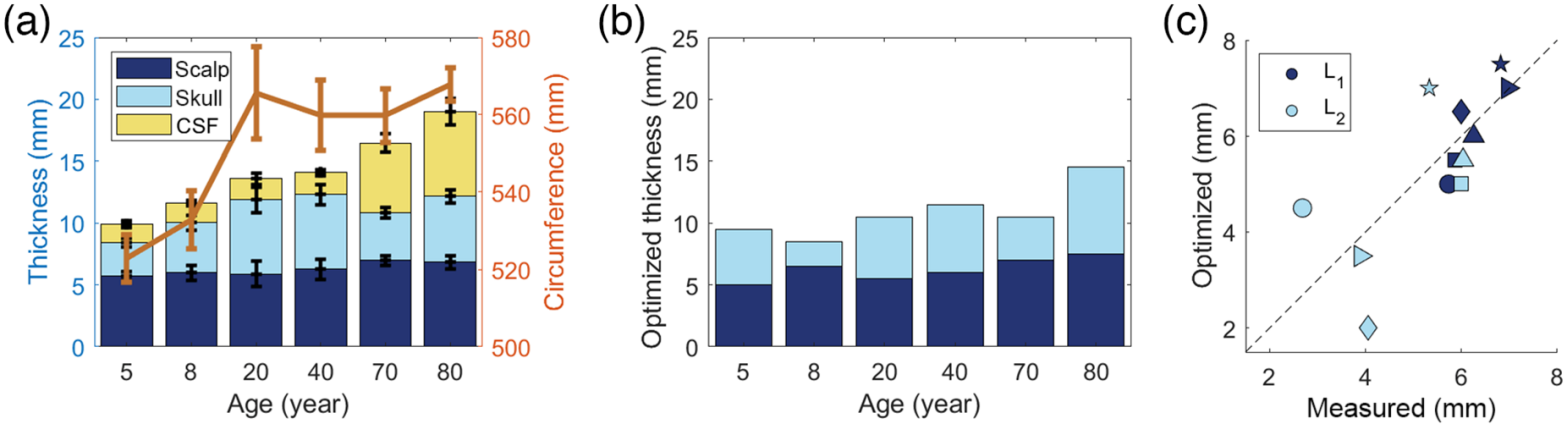
(a) Mean and standard deviation of the volume-averaged scalp (dark blue), skull (light blue), and CSF (yellow) thickness as well as head circumference (orange) across several axial slices near the source–detector plane on each MRI templates. (b) Optimized scalp and skull (light and dark blue, respectively) thickness obtained from pressure modulation. (c) Comparison of measured volume-averaged layer thickness with the optimized thickness estimate from pressure modulation for scalp ($L_{1}$, in dark blue) and skull ($L_{2}$, in light blue). Different shapes denote the six different MRI templates: circle is 5y, diamond is 8y, square is 20y, upper triangle is 40y, right triangle is 70y, and pentagram is 80y. Dotted line denotes the line of unity.

The volume-averaged measured thickness of the extracerebral layers in the MRI age-averaged templates increased with age, as expected [Fig. 6(a)]. These increases were driven by increases in skull thickness from 5 to 20 years, and by increases in CSF thickness from 40 to 80 years. Average head circumference sharply increased during adolescence and remained constant throughout adulthood. The standard deviation in the head circumference of 10 to 15 mm reflects the variation of head size across axial slices. Figure 6(b) shows the optimized thickness of the skull and scalp layers derived from the pressure modulation method outlined in Sec. 2.3. As shown in Fig. 6(c), the optimized scalp thickness is correlated with the volume-averaged measured scalp thickness, but the two measures can deviate by as much as 2 mm. In general, the optimized skull thicknesses deviate more from the volume-averaged skull thickness than the optimized/measured scalp thicknesses.

**Fig. 7:**
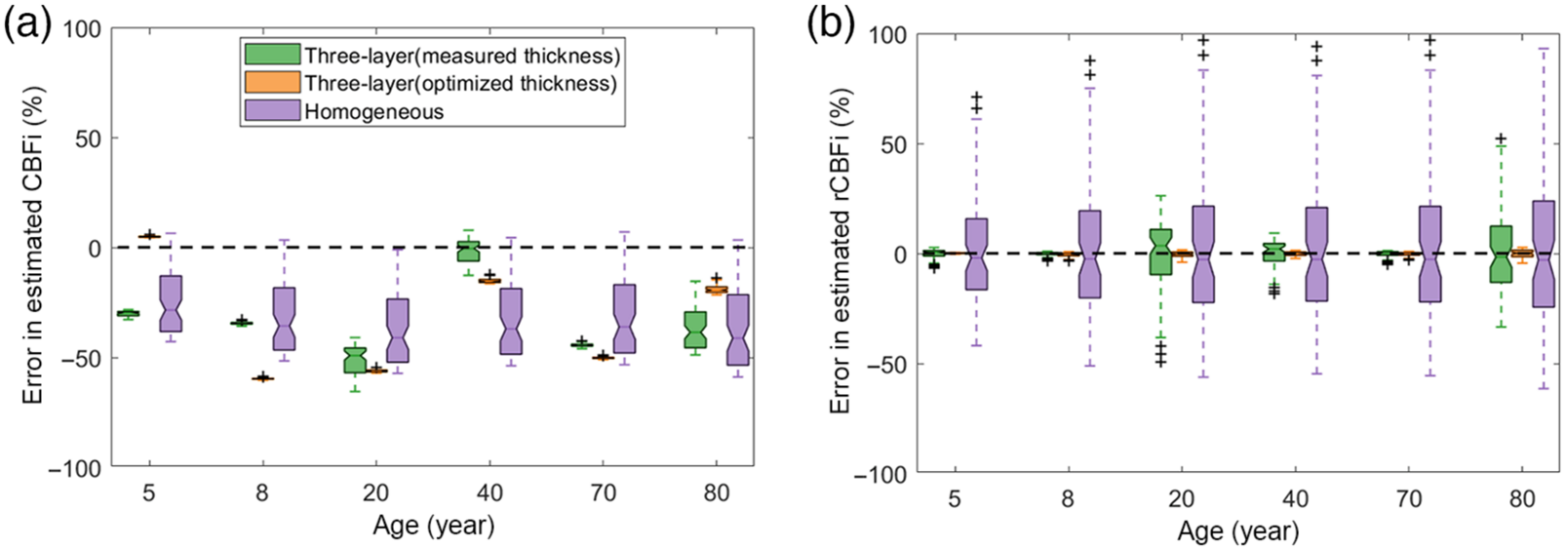
Comparison of three-layer and homogeneous models. Boxplots of the error in estimated CBFi (a) and relative change in CBFi (b) as a function of age. For each boxplot, the central line denotes the median, and the bottom and top edges of the box indicate the 25’th and 75’th percentiles, respectively, of the 72 samples tested. The whiskers extend to the most extreme data points not considered outliers.

As shown in Fig. 7(a), when using either the homogeneous or the 3-layer model to estimate CBFi from the MRI age-averaged templates, CBFi was typically significantly underestimated. The magnitude of this underestimation varied wildly as a function of age for the three-layer estimations, presumably due to heterogeneity in layer thickness that can have appreciable influence on estimation accuracy. For relative changes in CBFi, the median error across all simulated combinations of CBFi/SBFi was approximately zero. As expected, the variation in this error about the median was appreciably larger for the semi-infinite compared to the three-layer model. The three-layer model with optimized thickness has the smallest range of error in rCBFi across the CBFi/SBFi combinations tested [Fig. 7(b)].

## 4 Discussion

The three-layer model is designed to separate cerebral hemodynamics from extracerebral contaminations to the DCS measured signal. Although this model does not fully recapitulate the complex structure of the human head, it strikes a balance between model complexity and estimation accuracy, while providing a significant improvement over the traditional homogenous model, which suffers from substantial extracerebral contaminations. Despite these advantages, there are several key features that the model fails to incorporate, including CSF, head curvature, and layer heterogeneity. The findings herein demonstrate that these factors can lead to appreciable errors in the estimation of cerebral blood flow. However, relative changes in CBFi can be recovered in a manner that is relatively insensitive to these factors, suggesting that the three-layer model is a promising approach to improve brain sensitivity of relative changes in perfusion with DCS.

**Fig. 8:**
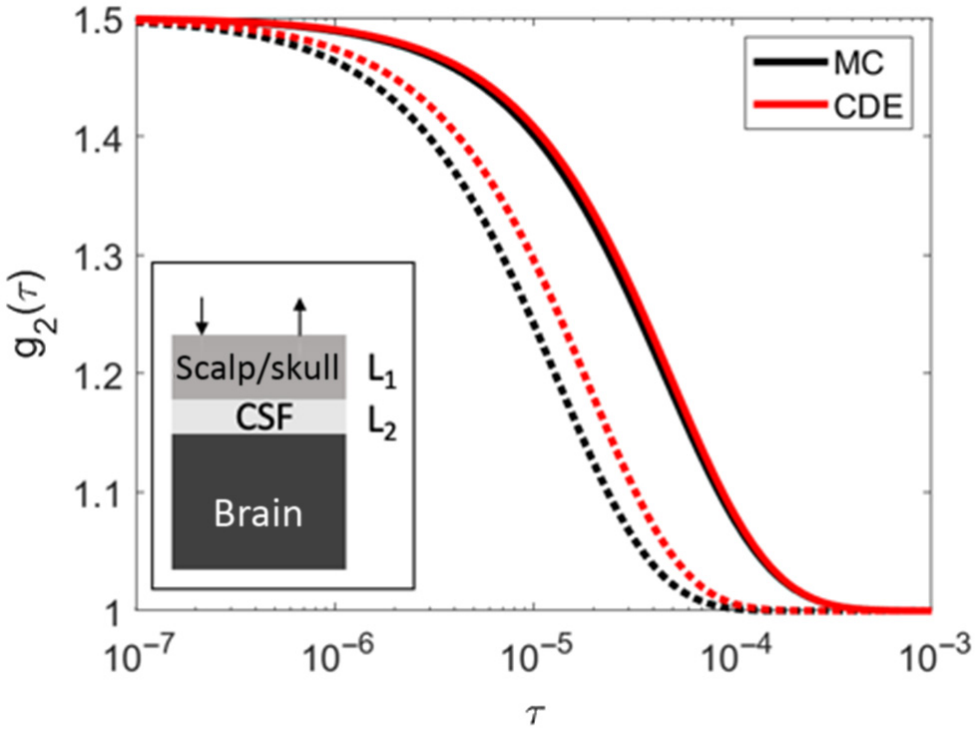
Effect of CSF. Comparison of intensity autocorrelation curves, $g_{2}(\tau )$, simulated using Monte Carlo (MC, black) and the correlation diffusion equation (CDE, red) at 1 cm (solid line) and 2.5 cm (dashed line) for a three-layer slab medium consisting of 10 mm scalp/skull (layer 1), 2 mm CSF (layer 2), and brain (layer 3).

Our results show that grouping CSF with the skull layer [Fig. 1(b)] leads to an overestimation of CBFi, while totally ignoring CSF [Fig. 1(c)] causes an underestimation of CBFi. In general, the correlation diffusion equation (CDE) breaks down in the presence of the very low absorption and scattering coefficient of the CSF. This breakdown can be visualized in Fig. 8 wherein we compare $g_{2}$ from a three-layered medium with a CSF as layer 2. Data were generated with MC and the three-layer solution to the CDE. The CDE generated curve is right shifted compared to the MC data. Thus, when CSF is grouped with the skull layer as layer 2 [Fig. 1(b)], using the CDE to fit MC-generated data in the presence of CSF leads to an overestimation of CBFi. However, the same overestimation was not found when CSF is grouped with the layer 3 [Fig. 1(c)]. We believe this contrary finding is because in the latter case, other factors also contribute to the error in CBFi. For one, the effective layer 3 flow in this case is a weighted combination of the zero flow CSF and brain flow, so we expect the estimated CBFi to be underestimated. Further, the effective optical properties of layer 3, which are a weighted combination of CSF and brain optical properties, are smaller than the values assumed in the fitting process. Overestimated layer 3 optical properties can have an appreciable effect on CBFi.^16^ In total, it appears these effects combine to lead to a net underestimation of CBFi, which agrees with a previous work investigating the influence of CSF with the two-layer model.^39^ We note that the influence of overestimated optical properties is not relevant in the former case when CSF is grouped in layer 2 because errors in layer 2 optical properties have minimal influence on CBFi estimation with the three-layer model.^16^

**Fig. 9:**
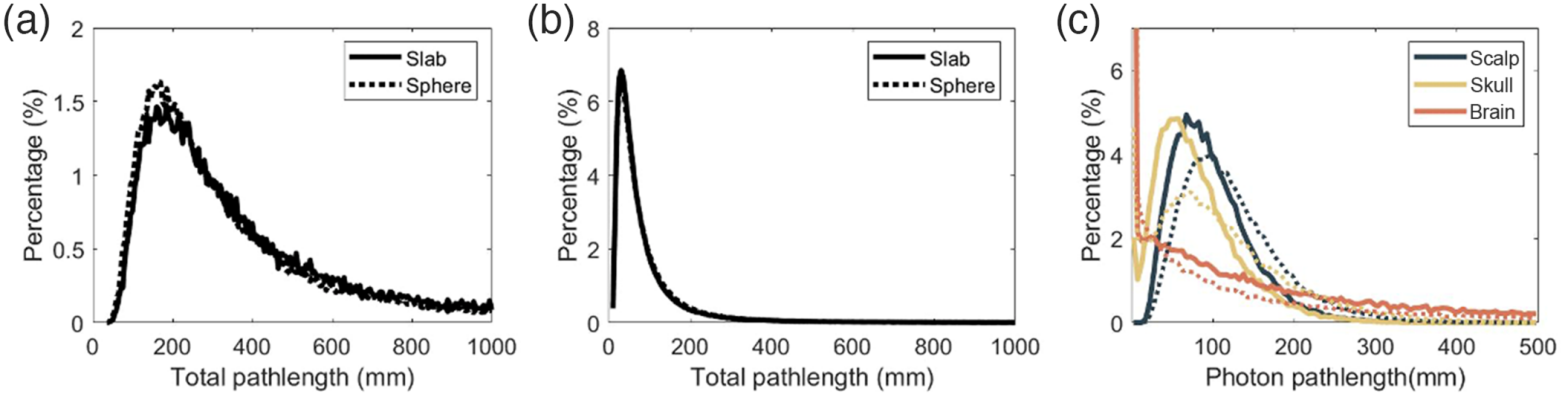
Distribution of the total pathlength of all detected photons at 1 cm (a) and 2.5 cm (b) for simulations performed on the sphere with radius 70 mm. (c) Distribution of pathlength of all detected photons at 2.5 cm in each layer (scalp in gray, skull in yellow, and brain in orange). In each subplot, the solid line denotes the three-layer slab and the dotted line denotes the three-layer sphere geometry.

Our results also show that curvature can lead to an underestimation of CBFi. Curvature can cause changes in the photon pathlength distribution,^40^ particularly for photons detected at 2.5 cm [Figs. 9(a) and 9(b)]. These changes are largely caused by a right-shift in the pathlength distribution within the scalp/skull and a slight left shift in the pathlength distribution in the brain [Fig. 9(c)]. The net effect of this distribution change is that the autocorrelation curves are slightly right shifted, which translates to an underestimation of CBFi. As was the case with CSF, these underestimations do not propagate to the error in rCBFi, thus the effect of curvature on rCBFi is minimal.

**Fig. 10:**
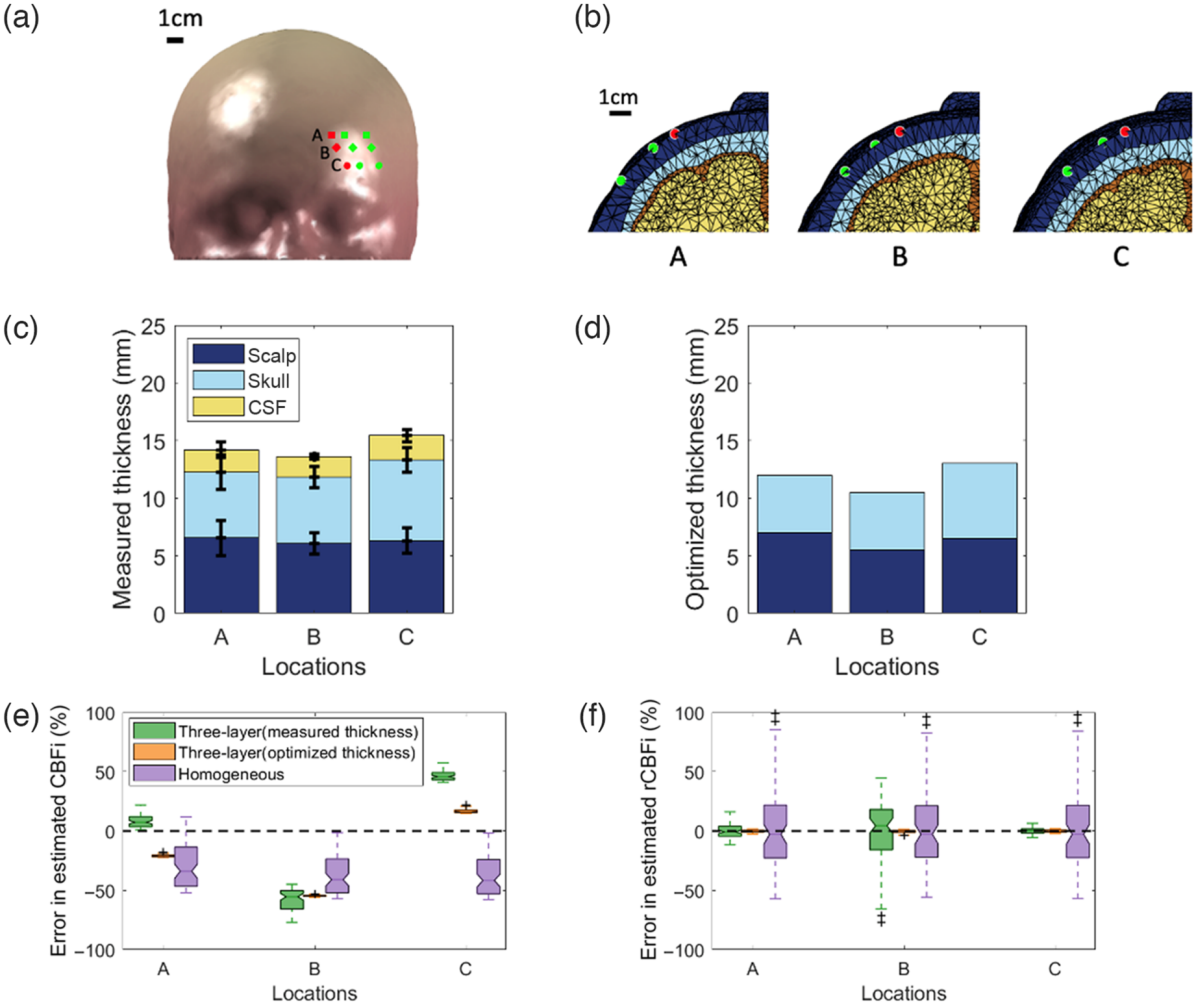
(a) Three different source–detector pair locations (A, B, C) on the surface of the forehead. (b) Visualization of the axial slices containing the source–detector pairs from panel (a). (c) Measured volume-averaged layer thicknesses (scalp in dark blue, skull in light blue, and CSF in yellow) and (d) optimized layer thickness for the three different locations of source–detector pairs shown in (a, b). (e) Boxplots of the error in estimated CBFi and (f) relative change in CBFi (rCBFi) at these locations using the three-layer model with measured thickness (green), optimized thickness (orange), and the homogeneous model (purple). For each boxplot, the central line denotes the median, and the bottom and top edges of the box indicate the 25’th and 75’th percentiles, respectively, of the 72 samples tested. The whiskers extend to the most extreme data points not considered outliers.

The simulations performed on the three-/and four-layer slab and sphere models allowed us to isolate the effects of curvature and CSF. In contrast, our modeling results from the MRI age-averaged templates provide insights into the cumulative effects of these factors on the accuracy of CBFi. Consistent with the results from Figs. 4 and 5, we found that CBFi was underestimated in all MRI-templates with the exception of 40 years. As shown in Fig. 10, the magnitude of these errors is highly influenced by small variations in the positions of the source and detector optodes. By varying the axial location of the optodes by ±1  cm, the error in CBFi varied from ∼−50% to 50% [Fig. 10(e)], demonstrating that the cumulative effects of curvature, CSF, and layer heterogeneity on the estimation accuracy of CBFi can be appreciable. We note that the investigation of optode position (Fig. 10) was performed in the 40Y template. We anticipate that as extracerebral thickness decreases, brain sensitivity of the model should increase, and as such, CBFi variability with optode positioning should be reduced.

To mitigate the errors in CBFi estimation due to the difficulty in defining layer thickness, we investigated an approach to identify an equivalent, “optimized” thickness via a pressure modulation. While this approach did not improve estimation accuracy of CBFi [Figs. 7(a) and 10(e)], it did significantly improve the estimation accuracy of rCBFi, as was suggested by Ref. 13. Thus, we recommend utilizing this pressure modulation approach to improve estimation accuracy of rCBFi when using the three-layer model. However, we note that pressure modulation *in vivo* may induce variations in scalp thickness that were not accounted for here and that could affect estimation accuracy in practice. Future research is needed to explore the validity of this pressure-modulation approach via an *in vivo* comparison against a gold standard perfusion technique.

There are several limitations in this study. Noise was not considered in our simulations; thus, the errors reported likely represent the best-case scenario. Moreover, we assumed optical properties for scalp, skull, and brain layers were known. Inaccuracies in these properties will likely compound estimation inaccuracies.^16^

## 5 Conclusion

We quantified the influence of curvature, CSF, and layer heterogeneity on the estimation of cerebral blood flow when using a three-layer model to analyze DCS data. These factors cause significant errors in CBFi; however, the influence of these factors on the estimation of relative changes in cerebral blood flow are minimal.

## Data and Code Availability Statement

The code and data used to generate figures is available at Github https://github.gatech.edu/Buckley-Lab-Code-Repository/Oversimplification_of_human_head.git.
